# Identification of Shaker Potassium Channel Family Members and Functional Characterization of *SsKAT1.1* in *Stenotaphrum secundatum* Suggest That *SsKAT1.1* Contributes to Cold Resistance

**DOI:** 10.3390/ijms25179480

**Published:** 2024-08-31

**Authors:** Dong-Li Hao, Jia Qu, Zhi-Yong Wang, Dao-Jin Sun, Sheng-Nan Yang, Jian-Xiu Liu, Jun-Qin Zong, Hai-Long Lu

**Affiliations:** 1 The National Forestry and Grassland Administration Engineering Research Center for Germplasm Innovation and Utilization of Warm-Season Turfgrasses, Jiangsu Key Laboratory for the Research and Utilization of Plant Resources, Institute of Botany, Jiangsu Province and Chinese Academy of Sciences (Nanjing Botanical Garden Mem. Sun Yat-Sen), Nanjing 210014, China; haodongli@jib.ac.cn (D.-L.H.); sundaojin@jib.ac.cn (D.-J.S.); turfunit@cnbg.net (J.-X.L.); 2 Sanya Nanfan Research Institute, College of Tropical Agriculture and Forestry, Hainan University, Haikou 570228, China; 22220953000028@hainanu.edu.cn (J.Q.); wangzhiyong@hainanu.edu.cn (Z.-Y.W.); 23210834000011@hainanu.edu.cn (S.-N.Y.)

**Keywords:** Shaker potassium channel, *KAT1*, cold resistance, salt tolerance, *Stenotaphrum secundatum*

## Abstract

*Stenotaphrum secundatum* is an excellent shade-tolerant warm-season turfgrass. Its poor cold resistance severely limits its promotion and application in temperate regions. Mining cold resistance genes is highly important for the cultivation of cold-resistant *Stenotaphrum secundatum*. Although there have been many reports on the role of the Shaker potassium channel family under abiotic stress, such as drought and salt stress, there is still a lack of research on their role in cold resistance. In this study, the transcriptome database of *Stenotaphrum secundatum* was aligned with the whole genome of Setaria italica, and eight members of the Shaker potassium channel family in *Stenotaphrum secundatum* were identified and named *SsKAT1.1*, *SsKAT1.2*, *SsKAT2.1*, *SsKAT2.2*, *SsAKT1.1*, *SsAKT2.1*, *SsAKT2.2*, and *SsKOR1*. The *KAT3*-like gene, *KOR2* homologous gene, and part of the AKT-type weakly inwardly rectifying channel have not been identified in the *Stenotaphrum secundatum* transcriptome database. A bioinformatics analysis revealed that the potassium channels of *Stenotaphrum secundatum* are highly conserved in terms of protein structure but have more homologous members in the same group than those of other species. Among the three species of *Oryza sativa*, *Arabidopsis thaliana*, and *Setaria italica*, the potassium channel of *Stenotaphrum secundatum* is more closely related to the potassium channel of *Setaria italica*, which is consistent with the taxonomic results of these species belonging to Paniceae. Subcellular location experiments demonstrate that SsKAT1.1 is a plasma membrane protein. The expression of *SsKAT1.1* reversed the growth defect of the potassium absorption-deficient yeast strain R5421 under a low potassium supply, indicating that *SsKAT1.1* is a functional potassium channel. The transformation of *SsKAT1.1* into the cold-sensitive yeast strain INVSC1 increased the cold resistance of the yeast, indicating that *SsKAT1.1* confers cold resistance. The transformation of *SsKAT1.1* into the salt-sensitive yeast strain G19 increased the resistance of yeast to salt, indicating that *SsKAT1.1* is involved in salt tolerance. These results suggest that the manipulation of *SsKAT1.1* will improve the cold and salt stress resistance of *Stenotaphrum secundatum*.

## 1. Introduction

*Stenotaphrum secundatum* is an excellent warm-season turfgrass that is widely used in tropical and subtropical areas because of its superior shade tolerance and feed value. Given the lack of excellent shade-tolerant grass species in temperate regions and the poor cold resistance of *Stenotaphrum secundatum* [1,2,3,4,5,6,7], it is particularly urgent to cultivate cold-resistant *Stenotaphrum secundatum* for use as an excellent shade-tolerant grass species in temperate regions. However, this cultivation depends on the mining of cold resistance genes in *Stenotaphrum secundatum*.

Studies have shown that the absorption of potassium by plants involves a high-affinity potassium absorption system composed of potassium (K^+^) transporters (*HAK*, *HKT*, and *CPA*), which play an important role when the external K^+^ concentration is <1 mM, and a low-affinity K^+^ absorption system composed of K^+^ channels (Shaker, *TPK*, and *Kir-like K^+^*) (which play an important role when the external K^+^ concentration is >1 mM) [8,9,10]. The Shaker K^+^ channel is the most widely studied gene family of plant potassium channels. Its family members have been identified in many plant species, including *Arabidopsis thaliana*, *Oryza sativa*, *Zea mays*, *Setaria italica*, *Gossypium hirsutum*, and other plants [11,12,13,14,15]. In *Arabidopsis thaliana*, Shaker K^+^ can be divided into five categories—I: *AKT* inward rectifier channel (*AtKT1*, *AtKT5*, and *AtSPIK*); II: *KAT* inward rectifier channel (*AtKAT1* and *AtKAT2*); III: *AKT* weak inward rectifier channel (*AtKT2/3*); IV: regulatory subunits involved in inward rectifier conductance formation (*AtKAT3*); and V: outward rectifier channel (*AtGORK* and *AtSKOR*) [8,16]. *AtKT1* mediates the absorption of potassium from the soil solution by roots [17]. *AtKT2/3* is expressed mainly in the phloem and xylem of the aboveground parts of plants. Its role is to activate the plasma membrane of phloem cells through electrical energy to promote the downwards transportation of photosynthetic sugars and facilitate the efficient loading of sugars [18]. *AtKT5* and *AtSPIK* are expressed in pollen and play a role in ensuring potassium nutrition in pollen [19,20]. *AtKAT1*, *AtKAT2*, and *AtGORK* are expressed in guard cells and coordinate with each other to regulate stomatal movement [17]. *AtKAT3* does not form functional potassium channels by itself but rather downregulates the activity of *AtKT1* and *AtKAT1* channels by forming heterotetramers with *AtKT1* or *AtKAT1* [11]. Highly conserved and specific sequences make Shaker K^+^ channels highly ion-selective. They all contain a cytosolic N-terminus, a cytosolic C-terminus, and six transmembrane α helices called S1-S6. The C-terminus includes a cyclic nucleotide binding domain (CNBD), an ankyrin repeat (Ank, contained by some family members), and a dimerization domain (KHA) of potassium channels, which function as homologous or heterotetramers [12,17,21]. The role of Shaker K^+^ channels in the plant abiotic stress response has been studied in many plant species. In *Oryza sativa*, some Shaker K^+^ channel members are involved in stomatal opening and closing under drought stress [22,23]. The overexpression of *OsAKT1* and *OsKAT1* in *Oryza sativa* can increase the tissue K^+^ content and improve plant drought and salt tolerance [13,24]. The overexpression of the *GmAKT1* gene in *Arabidopsis thaliana* increases root length and plant K^+^ concentration under drought and salt stress [25]. Taken together, although there have been many reports on the role of Shaker K^+^ channel family plants in response to abiotic stresses such as drought and salt stress, research on their role in cold resistance is still scarce [26]. Since potassium enhances plant cold tolerance, the Shaker K^+^ channel, which mediates potassium absorption, may play a role in plant cold resistance. Therefore, in addition to the reported transcription factors and metabolite synthases (*UGTs*, *NCED*, *RafS*, *NAC*, and *DREB/CBF*) [27,28,29,30,31,32], the Shaker K^+^ channel may be a novel cold resistance gene.

In this study, we compared the transcriptome database of *Stenotaphrum secundatum* with the whole genome of *Setaria italica* to identify the members of the Shaker K^+^ channel gene family of *Stenotaphrum secundatum*. Through the analysis of basic physical and chemical properties, protein two-dimensional and three-dimensional structures, and phylogenetic trees, the protein characteristics and evolutionary relationships of the Shaker K^+^ channel in *Stenotaphrum secundatum* were determined. *SsKAT1.1* was subsequently transferred into the potassium absorption-deficient yeast strain R5421 to determine its potassium absorption ability. *SsKAT1.1* was also transferred into the low-temperature-sensitive yeast INVSC1 to study its role in cell cold resistance. *SsKAT1.1* was finally transferred into the salt-sensitive yeast G19 to study its role in cell salt tolerance. In this study, we identified the biological information of the members of the Shaker K^+^ channel family of *Stenotaphrum secundatum* and the potential role of *SsKAT1.1* in potassium nutrition, cold resistance, and salt tolerance. This study provides a foundation for breeding a new variety of cold stress-resistant *Stenotaphrum secundatum* strains via the manipulation of *SsKAT1.1*.

## 2. Results

### 2.1. Gene Identification

Through BLAST comparison with the Shaker K^+^ channel gene family of *Setaria italica*, nine candidate genes for the Shaker K^+^ channel were preliminarily screened from the transcriptome database of *Stenotaphrum secundatum*. There were four *KAT*-type inward rectifier channels: *SsKAT1.1*, *SsKAT1.2*, *SsKAT2.1*, and *SsKAT2.2*; two *AKT*-type inward rectifier channels: *SsAKT1.1* and *SsAKT1.2*; two *AKT*-type weak inward rectification channels: *SsAKT2.1* and *SsAKT2.2*; and one outward rectifier potassium channel: *SsKOR1*. The CDS length of these genes ranged from 1302 to 2559 bp, and the number of encoded amino acids ranged from 433 to 852 (Table 1). Compared with that of the homologous *SsAKT1.1* (716 amino acids), the number of amino acids encoded by *SsAKT1.2* (433 amino acids) is much smaller, indicating that it may not be a Shaker K^+^ channel.

### 2.2. Motif and Protein Domain Analysis

**Figure 1:**
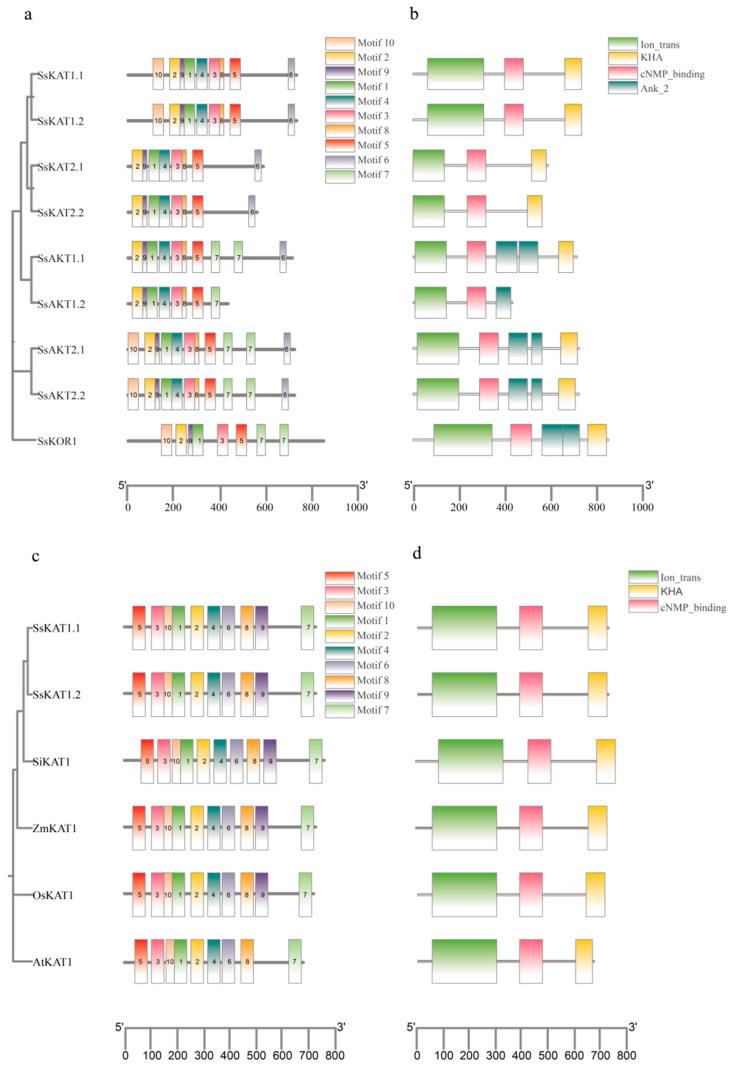
**Motif and domain of Shaker K+ channel.** (**a**) Motif analysis of Shaker K^+^ channel proteins in *Stenotaphrum secundatum*. (**b**) Analysis of 9 Shaker K^+^ channel protein domains in *Stenotaphrum secundatum*. (**c**) Motif analysis of *SsKAT1*, *AtKAT1*, *OsKAT1*, *SiKAT1*, and *ZmKAT1*. (**d**) Protein domain analysis of *SsKAT1*, *AtKAT1*, *OsKAT1*, *SiKAT1*, and *ZmKAT1*. Notably, the motifs in (**a**,**c**) have different sequences, although they have the same name.

The motif and protein domain analysis of the *Stenotaphrum secundatum* Shaker K^+^ potassium channel is shown in Figure 1a,b. Compared with other candidate genes, *SsAKT1.2* lacks the key KHA protein domain in the potassium channel (Figure 1b), and the number of encoded amino acids is too short (Table 1), suggesting that *SsAKT1.2* is not a member of the Shaker K^+^ channel. Therefore, *SsAKT1.2* was excluded from the rest of this study. Among the candidate genes, there was no difference in conserved motifs between homologues (*SsKAT1*, *SsKAT2*, *SsAKT2*). Compared with the inward rectifier channel, the outward rectifier channel *SsKOR1* lacks motif 6 at the C-terminus. *SsKAT2* and *SsAKT1* have high similarity at the C-terminus. *SsKAT2* and *SsAKT1* have high similarity at the N-terminus, as do *SsKAT1* and *SsAKT2* (Figure 1a). Compared with other candidate genes, *SsKAT1* and *SsKAT2* lack two of motif 7, which is consistent with the lack of two Ank2 in the protein domain (Figure 1a,b). Given that the other four published *KAT1* genes also lack two Ank2 domains (Figure 1d), it is likely that the Ank2 domain is not necessary for some potassium channels. On the basis of the above analysis, eight candidate genes, *SsKAT1.1*, *SsKAT1.2*, *SsKAT2.1*, *SsKAT2.2*, *SsAKT1.1*, *SsAKT2.1*, *SsAKT2.2*, and *SsKOR1*, were identified as Shaker K^+^ channels.

Motif and protein domain analyses of *KAT1* proteins from five species are shown in Figure 1c,d. Compared with the other four published *KAT1* genes, *SsKAT1* has no difference in its protein domain, and its function may be similar to that of these four genes (Figure 1d). Notably, compared with other homologous genes, *AtKAT1* lacks motif 9 (Figure 1c). The presence or absence of motif 9 suggests that the functional performance of *SsKAT1* may differ from that of *AtKAT1*.

### 2.3. Protein Properties

The results of ProtParam revealed that the molecular weight of a single protein encoded by the Shaker K^+^ channel gene of *Stenotaphrum secundatum* ranged from 63.98 kDa to 96.56 kDa. The isoelectric point (pI) of the protein ranged from 6.00 to 8.06. *SsKOR1*, *SsKAT1.1*, *SsKAT1.2*, *SsAKT1.1*, *SsAKT2.1*, and *SsAKT2.2* are acidic; *SsKAT2.1* and *SsKAT2.2* are basic. The eight Shaker K^+^ channel proteins are hydrophilic (GRAVY < 0). Except for *SsKOR1*, the instability coefficients of the other seven channel proteins are less than 40, indicating that the proteins are relatively stable (Table 2).

### 2.4. Protein Secondary Structure and Three-Dimensional Structure Prediction

**Figure 2:**
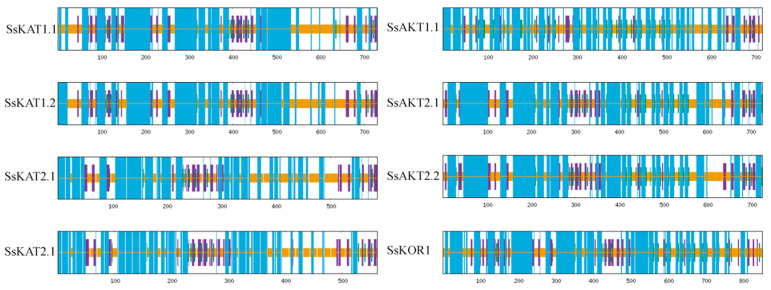
**Protein secondary structure prediction.** Blue represents the alpha helix, brown represents the extended strand, green represents the beta turn, and the random coil is purple.

The distribution of secondary structures among protein homologies (*SsKAT1*, *SsKAT2*, and *SsAKT2*) is similar. The secondary structures are divided into four types: an α helix, β-turn, random coil, and extended strand. The proportion of α helices in all protein channels is more than 40%, and the content of α helices in *SsKOR1* is the highest, which is 55.4%. A random coil plays a role in connecting other structures. An extended strand is mainly distributed in the C-terminal domains, and a β-turn is the least prevalent. The results show that the α helix of *SsKAT1* is mainly distributed in the range of the first 400 amino acids (Table 3, Figure 2).

**Figure 3:**
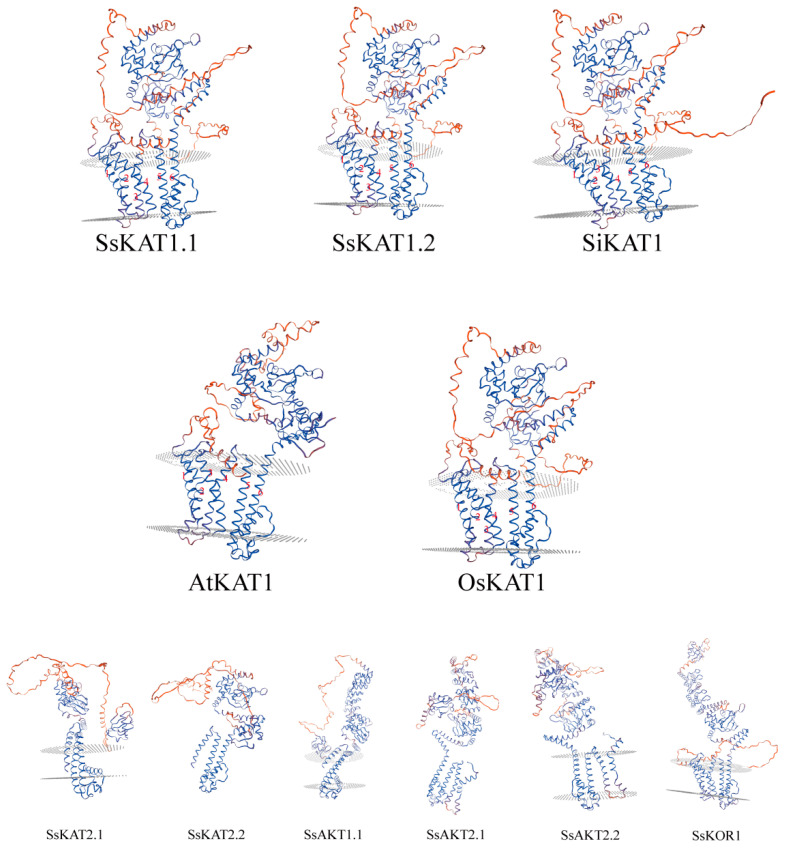
**Protein three-dimensional structure prediction.** The three-dimensional structures of 8 Shaker K+ potassium channels from *Stenotaphrum secundatum* and *KAT1* from *Arabidopsis thaliana*, *Setaria italica*, and *Oryza sativa* are presented. The blue ribbons represent areas with more accurate predictions, whereas the red ribbons represent areas with lower prediction accuracy. A view from top to bottom: the intracellular region, transmembrane region, and extracellular region. Six transmembrane structures are labelled with Arabic numerals. All the modelling results have a GMQE > 0.7.

Swiss-model modelling results show that the eight Shaker K^+^ channels of *Stenotaphrum secundatum* have a large number of α helix structures, and three-dimensional structure results of homology (*SsKAT1*, *SsKAT2*, and *SsAKT2*) are similar. Like *AtKAT1*, *SiKAT1*, and *OsKAT1*, *SsKAT1* also predicts six transmembrane structures, which is consistent with the theory of six transmembrane α helices plus a large cnmp_binding in a Shaker K^+^ channel. The three-dimensional structure of *SsKAT1* is more similar to *SiKAT1* and *OsKAT1*, but different to *AtKAT1* (Figure 3).

### 2.5. Analysis of the Phylogenetic Relationship of the Shaker K+ Channel

**Figure 4:**
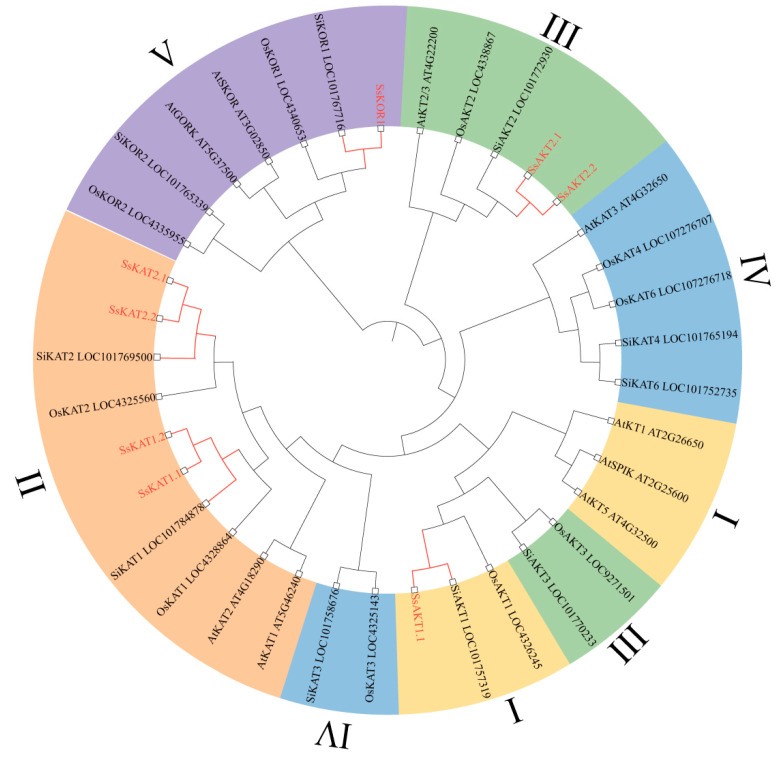
Shaker K^+^ channel phylogenetic tree constructed from proteins from *Stenotaphrum secundatum*, *Arabidopsis thaliana*, *Oryza sativa*, and *Setaria italica*. Shaker K^+^ channels are divided into five categories—I: *AKT*-type inward rectifier channel (yellow); II: *KAT*-type inward rectifier channel (brown); III: *AKT*-type weak inward rectifier channel (green); IV: regulatory subunit involved in formation of inward rectifier conductance (blue); and V: outward rectifier channel (purple).

According to the results of the phylogenetic tree, Shaker K^+^ channels are divided into five categories—I: *AKT*-type inward rectifier channel (yellow); II: *KAT*-type inward rectifier channel (brown); III: *AKT*-type weak inward rectifier channel (green); IV: regulatory subunit involved in the formation of inward rectifier conductance (blue); and V: outward rectification channel (purple). In this study, a total of eight Shaker K+ channels were identified: *SsAKT1.1* belongs to class I; *SsKAT1.1*, *SsKAT1.2*, *SsKAT2.1*, and *SsKAT2.2* belong to class II; *SsAKT2.1* and *SsAKT2.2* belong to class III; and *SsKOR1* belongs to class V. The published family members of *Arabidopsis thaliana* (9), *Oryza sativa* (10), and *Setaria italica* (10) are distributed in all five classifications. Notably, the *KAT3*-like protein, KOR2 homologous gene, and part of the *AKT*-type weakly inwardly rectifying channel have not been identified in *Stenotaphrum secundatum*. Specifically, compared with those in millet and *Oryza sativa*, *KOR2*, *KAT3*, *KAT4*, *KAT6*, and *AKT3* corresponding genes have not been identified in the transcriptome database of *Stenotaphrum secundatum*. When compared with those of *Arabidopsis thaliana*, no corresponding genes of *KAT3*, *KT2/3*, *KT5*, *SPIK*, *SKOR*, or *GORK* were identified (Figure 4).

### 2.6. Relative Expression Levels of Shaker K^+^ Channel Family Members in Different Tissues

**Figure 5:**
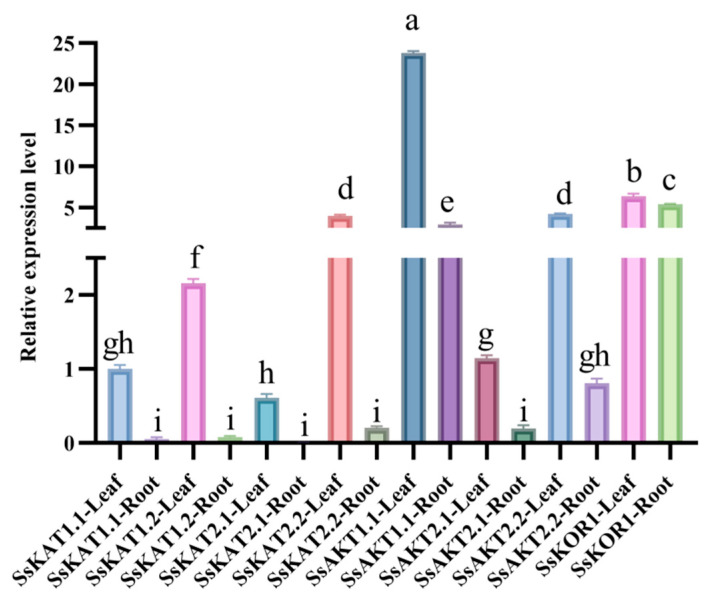
Expression patterns of Shaker K^+^ channels in the root and the leaf of *Stenotaphrum secundatum.* Significant differences among treatments are indicated by different letters (*p* < 0.05).

Real-time quantitative PCR (qPCR) was used to study the expression patterns of genes in different tissues (root or leaf) of *Stenotaphrum secundatum*. Except for the extremely low expression level of *SsKAT2.1* in the root, all other family members are expressed in both the root and the leaf, and there are differences in expression levels between different genes in the same tissue and between different tissues of the same gene (Figure 5). All Shaker K^+^ channel genes have higher leaf expression levels than in the root. Compared with the great expression level gaps between the two tissues in most genes, the expression differences of *SsKOR1* between different tissues are relatively small. In both the leaf and the root, *SsAKT1.1* has the highest expression level and *SsKAT2.1* has the lowest expression level (Figure 5).

### 2.7. SsKAT1.1 Is Located to Plasma Membrane

**Figure 6:**
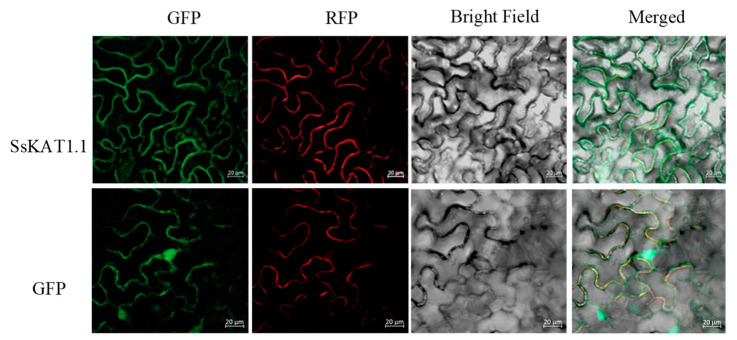
**The subcellular localization of the *SsKAT1.1* in the epidermis of tobacco.** The green fluorescence, red fluorescence, bright field, and merged views of tobacco epidermic cells expressing SsKAT1.1:GFP (**top**) or the 35S:GFP control (**below**) are accordingly indicated. Bar = 20 µm.

To determine the subcellular localization of *SsKAT1.1*, *SsKAT1.1* was introduced into tobacco cells using an agrobacterium-mediated transformation method. A plasma membrane marker was employed to indicate the position of the cell membrane. The results demonstrated that, consistent with previous reports, the empty vector localized to both the cell membrane and nucleus [33]. The localization of *SsKAT1.1* within the cell coincided with the position of the membrane marker, where green and red colours merged to form the yellow colour, indicating that *SsKAT1.1* is located on the cell plasma membrane (Figure 6).

### 2.8. SsKAT1.1 Has a Potassium Absorption Function

**Figure 7:**
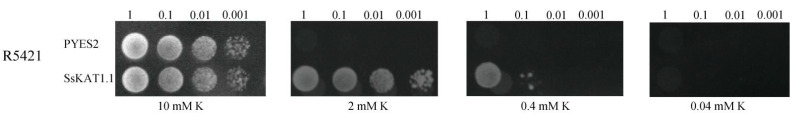
**The expression of *SsKAT1.1* in the potassium absorption-deficient yeast strain R5421.** The growth performance of empty vector- and *SsKAT1.1*-expressing yeast on AP media supplemented with different concentrations of KCl. The dilution factor of the yeast cells is indicated.

To clarify the potassium uptake function of *SsKAT1.1*, the gene was transformed into the potassium uptake-deficient yeast strain R5421. Under the condition of sufficient potassium supply (10 mM), the empty vector- and *SsKAT1.1*-expressing yeast strains grew normally without significant differences, indicating that the expression of different plasmids did not affect the normal growth of yeast (Figure 7). Under a limited potassium supply (≤5 mM), the empty vector-expressing yeast could not survive, which was similar to a previous report [15]. The expression of *SsKAT1.1* rescued the growth defect under 2 mM K^+^. With decreasing external potassium concentration (from 2 mM to 0.4 mM), the supporting effect of *SsKAT1.1* on the growth of potassium absorption-deficient yeast decreased. Under 0.04 mM K^+^ supply, the performance of *SsKAT1.1* was similar to that of the empty vector, and none of the yeast survived. The above results show that *SsKAT1.1* has a potassium absorption function and is dependent on the external potassium supply in a concentration-dependent manner.

### 2.9. Expression of SsKAT1.1 in Yeast Enhances Cold Resistance

**Figure 8:**
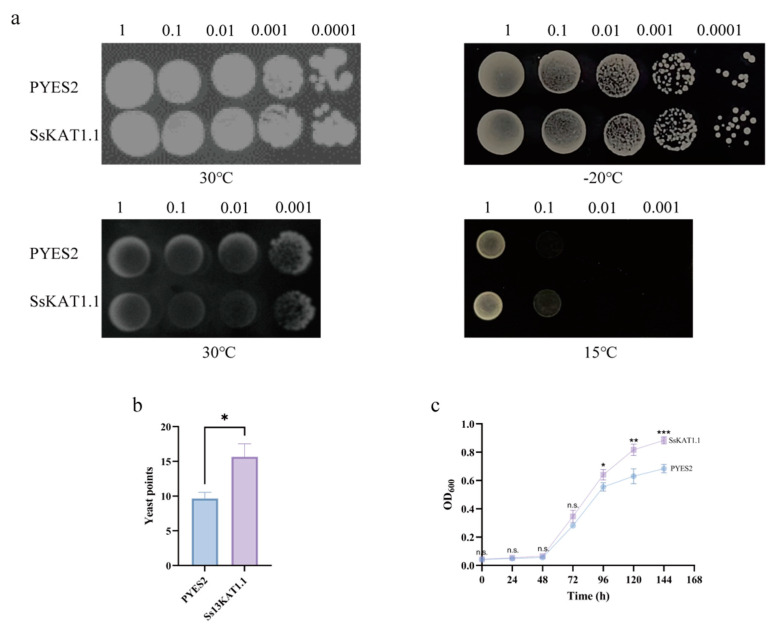
**The expression of *SsKAT1.1* in the cold-sensitive yeast strain INVSC1.** (**a**). The top panel shows the growth performance of yeast expressing the empty vector (pYES2) or *SsKAT1.1* on the medium with (**left**) or without (**right**) a freezing pretreatment at −20 °C. The below panel shows the growth performance of yeast expressing the empty vector (pYES2) or *SsKAT1.1* on the medium at different temperatures. The dilution factor of the yeast cells is indicated. (**b**). The statical analysis of survival numbers of yeast clones post −20 °C treatment. (**c**). The time dependence of OD600 of yeast expressing *SsKAT1.1* or the empty vector during liquid cultivation at 10 °C. Significant differences between treatments are indicated by asterisks (*p* < 0.05, *; *p* < 0.01, **; *p* < 0.001, ***). The n.s. indicates no significance.

To study the role of *SsKAT1.1* in freezing resistance, the growth performance of the cold-sensitive yeast strain INVSC1 harbouring *SsKAT1.1* or an empty vector with or without a freezing pretreatment was investigated. The results showed that under normal yeast culture temperature (30 °C), no significant difference in growth performance between the *SsKAT1.1*-expressing yeast and the empty vector-expressing yeast was detected. However, after pretreatment at −20 °C for 24 h, the *SsKAT1.1*-expressing yeast had better survival than empty vector-expressing yeast, indicating that *SsKAT1.1* plays a role in improving the freezing resistance of yeast cells (Figure 8a, top; Figure 8b).

To study the role of *SsKAT1.1* in chilling resistance, the growth performance of the cold-sensitive yeast strain INVSC1 harbouring *SsKAT1.1* or an empty vector at different temperatures was investigated. The results showed that the growth of the two groups was similar at 30 °C. When the temperature was reduced to 15 °C, the growth of *SsKAT1.1*-expressing yeast was better than that of the empty vector-expressing yeast (Figure 8a, below). Additionally, the OD600 of INVSC1 harbouring SsKAT1.1 is higher than that of yeast expressing the empty vector during liquid cultivation at 10 °C (Figure 8c). Both results indicate that *SsKAT1.1* functions in improving the chilling resistance of yeast cells. Since cold stress is composed of freezing stress and chilling stress, these results together suggest that *SsKAT1.1* contributes to the cold resistance of yeast cells.

### 2.10. Expression of SsKAT1.1 in Yeast Enhances Salt Tolerance

**Figure 9:**
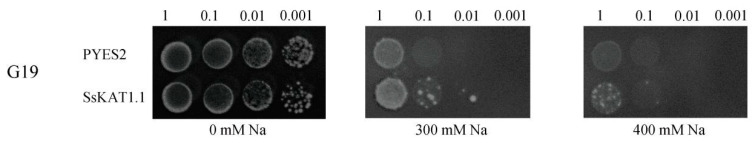
**The expression of *SsKAT1.1* in the salt-sensitive yeast strain G19.** The growth performance of empty vector- and *SsKAT1.1*-expressing yeast on AP media supplemented with different concentrations of NaCl. The dilution factor of the yeast cells is indicated.

The growth of the salt-sensitive yeast strain G19 harbouring an empty vector or *SsKAT1.1* under different concentrations of NaCl was then investigated. No significant difference in growth was detected between the two yeast strains in media without Na^+^, indicating that the normal growth of yeast was not affected by the transformation of different plasmids. Similar to previous reports, the growth of empty vector-expressing yeast was significantly inhibited by high concentrations of Na^+^ [34], while the expression of *SsKAT1.1* significantly increased the resistance of yeast to high concentrations of Na^+^ (Figure 9). These results demonstrate that the expression of *SsKAT1.1* enhances salt tolerance.

## 3. Discussion

### 3.1. Stenotaphrum secundatum and Setaria italica Shaker K^+^ Channels Have a Close Phylogenetic Relationship

In the phylogenetic tree, members of the Shaker K^+^ channel family in *Stenotaphrum secundatum* are classified in the same cluster as the homologous genes corresponding to *Setaria italica* (red tree branch in Figure 4), whereas they are classified with a far relationship with other Shaker K^+^ channel family members from model plant *Arabidopsis thaliana* and *Oryza sativa*. These results are conformed to the fact that the *Stenotaphrum secundatum* and *Setaria italica* both belong to the Paniceae family (https://www.iplant.cn/info/Trib.%20Paniceae?t=z, accessed on 27 August 2024). Compared with conventional model plants such as *Arabidopsis thaliana* and *Oryza sativa*, the use of *Setaria italica* as a model plant to identify the Shaker K^+^ channel in *Stenotaphrum secundatum* is more suitable. Since the genome data of *Setaria italica* have been published [15] and the genomic data of *Stenotaphrum secundatum* have not yet been released, the use of the genome data of *Setaria italica* to obtain homologous genes in *Stenotaphrum secundatum* is useful. The method of conducting a gene family analysis on the basis of the genomes of closely related species in plant taxonomy presented in this study provides a technical reference for subsequent related research.

### 3.2. Shaker K^+^ Channel Members of Stenotaphrum secundatum Have More than One Corresponding Gene

Compared with the tested plants, more than one corresponding gene was identified in *KAT1*, *KAT2*, and *AKT2* of *Stenotaphrum secundatum* (Figure 4). The plant genomes published by the NCBI are mostly artificially cultivated diploid homozygous or haploid varieties, such as *Arabidopsis thaliana* thale cress, *Oryza sativa* Japonica Group, and *Setaria italica* foxtail millet. Therefore, it is unlikely that there is more than one corresponding gene in these plant genomes. The *Stenotaphrum secundatum* used for transcriptional sequencing in this study was collected from the wild and not artificially homozygous, so it is highly likely that it is heterozygous, leading to the identification of more than one corresponding gene. Although *Stenotaphrum secundatum* is propagated mostly by creeping stems (asexual reproduction), genetic mutations still occur [35,36], further supporting the above speculation.

### 3.3. Several Shaker K^+^ Channels in Stenotaphrum secundatum Have Not Been Identified

According to the evolutionary tree, the *KAT3*-like gene, *KOR2* homologous genes, and some *AKT* weak inward rectifying channels were not identified in *Stenotaphrum secundatum* (Figure 4). This phenomenon may be due to the following reasons. As for *KAT3*, *AtKAT3* (also known as *AtKC1*) in *Arabidopsis thaliana*, along with the *KAT3*, *KAT4*, and *KAT6* genes in *Oryza sativa* and *Setaria italica*, is a regulatory subunit formed by inward rectifying conductance and is a type of protein that regulates the Shaker K^+^ channel rectifying channel [11,12,37,38,39,40]. The *AtKAT3* homologous gene *KZM2* in corn is expressed at significantly lower levels in plant tissues than the *AtKAT1* homologous gene *KZM3* [12]. *Oryza sativa* and *Solanum tuberosum KAT3* corresponding genes also have similar situations [41,42]. In *Gossypium hirsutum* and *Oryza sativa*, the expression levels of these genes significantly increase at specific times under certain stress conditions [14,41], which is consistent with the view that the expression of some K^+^ channels is only induced under stress [17,43]. In summary, the expression levels of *SsKAT3* may be extremely low, resulting in the inability to detect them in the transcriptome.

In terms of *KOR2* genes, *Oryza sativa OsKOR1* (formerly known as *OsGORK*) is expressed in most tissues, whereas *OsKOR2* (formerly known as *OsSKOR*) is expressed mainly in root vascular tissue, flowers, and the seed scutellum [44]. The sampling site for transcriptome sequencing in this study was leaves, which do not highly express *KOR2* genes, resulting in a failure to detect this gene in *Stenotaphrum secundatum*.

For genes such as *AKT2/3*, the *AKT* weak inward rectifying channel *AtKT2/3* in *Arabidopsis thaliana* is not only an *AKT2* gene but also an *AKT3* gene [45], the expression site of which is mainly in the phloem and xylem of the aboveground parts [46]. In *Ipomoea batatas* and *Setaria italica*, although the expression level of *AKT* weak inward rectifying channels is increased under certain stresses, the expression level is very low in most cases [15,17]. The failure of the *AKT3*-type gene to be identified in the transcriptome of *Stenotaphrum secundatum* may be due to the following reasons. First, the expression of *SsAKT2* in the Shaker K^+^ channel meets the needs of *Stenotaphrum secundatum*, resulting in the nontranscription or low transcription of *AKT3*-type genes. Second, the identified *SsAKT2* gene is similar to *AtKT2/3* in that it is both an *AKT2*-type gene and an *AKT3*-type gene. Third, *AKT3*-type genes in *Stenotaphrum secundatum* are also a type of gene that can be induced to be expressed only under certain stress conditions.

### 3.4. SsKAT1.1 Has a Potassium Absorption Function, and Its Potassium Absorption Function Depends on Higher Concentrations of K^+^

The expression levels of *SsKAT1.1*, *SsKAT1.2*, *SsAKT2.1*, and *SsAKT2.2* in the leaf of *Stenotaphrum secundatum* are much higher than those in the root (Figure 5), which is similar to the expression patterns of homologous genes in soybean and Arabidopsis. It is thus speculated that members of the class II family in the Shaker K^+^ channel family of *Stenotaphrum secundatum* may contribute to potassium nutrition in the leaf and root, like those in Arabidopsis [17,25]. The expression pattern of *SsKOR1* is similar to *GmGORK* and *OsKOR1*, all of which are expressed in both the leaf and the root [44]. The expression pattern of *SsAKT1.1* is similar to *SiAKT1* and *GmAKT1*, all of which have a high expression level in both the root and the leaf [15,25] (Figure 5). The differences in expression patterns among Shaker K^+^ channels may reflect their different division of labour in potassium nutrient uptake in different tissues.

The Ion_trans protein domain exists in sodium, potassium, and calcium channel proteins with six transmembrane α helical structures (https://www.ebi.ac.uk/interpro/entry/InterPro/IPR005821/protein/UniProt/#table, accessed on 27 August 2024); *SsKAT1.1* predicted the Ion_trans protein domain within the first 400 amino acid ranges, which is consistent with the distribution of α helices, mainly in the first 400 amino acids and in the secondary structure, and the prediction of six transmembrane α helices in three-dimensional modelling (Figure 1, Figure 2 and Figure 3). Moreover, *SsKAT1.1* has no domain differences from other homologous *KAT1* genes (Figure 1b, Table 2), so it is highly likely that *SsKAT1.1* is a *KAT1*-type gene with a potassium absorption function.

According to the subcellular location and yeast functional complementarity test (R5421), *SsKAT1.1* is a plasma membrane-located *KAT1* potassium ion channel with a potassium absorption function (Figure 6 and Figure 7). However, under the condition of 0.04 mM K^+^, the *SsKAT1.1*-expressing yeast strain did not survive, which is similar to the performance of the homologous *KZM3* (i.e., *ZmKAT1*) in *Zea mays* [12]. However, the above results differ from the normal growth of the *AtKAT1*-expressing yeast strain under 0.05 mM K^+^ conditions [47]. Compared with *SsKAT1.1* and *ZmKAT1*, *AtKAT1* lacks motif 9 (Figure 1b), which once again indicates that the presence or absence of motif 9 may lead to differences in functional characterization between *KAT1* homologous proteins.

### 3.5. SsKAT1.1 Participates in Cellular Cold Resistance

INVSC1 is often used to verify the cold resistance function of metabolite synthases (*UGTs*, *NCED*, and *RafS*) and transcription factors (*NAC* and *DREB/CBF*) [27,28,29,30,31,32]. In this study, we used INVSC1 for the first time to verify the cold resistance function of potassium ion channels and found that *SsKAT1.1* can increase cold resistance in cells (Figure 8). Currently, reports on cold resistance genes focus more on metabolite synthases and transcription factors (*UGTs*, *NCED*, *RafS*, *NAC*, and *DREB/CBF*), and the Shaker K^+^ channel identified in this study is a novel member of the cold resistance gene family.

### 3.6. SsKAT1.1 Participates in Cell Salt Tolerance

Maintaining appropriate K^+^/Na^+^ ratios is crucial for plant salt resistance, which is closely related to potassium ion channels [48]. G19, a salt-sensitive yeast, is commonly used to verify the salt resistance of plant potassium ion channels and potassium transporters [34,49,50,51]. *SsKAT1.1* showed similar results to that of its homologous gene *OsKAT1* when expressed in salt-sensitive yeast (Figure 9). Overexpressing *OsKAT1* in *Oryza sativa* results in greater potassium absorption ability to cope with salt stress [13]. Due to the limitations of farmland policies, turfgrass cannot occupy farmland resources, and its planting sites can only transfer to marginal soils with poor site conditions, such as saline–alkali soil. The *SsKAT1* gene identified in this study provides genetic resources for the cultivation of salt-tolerant *Stenotaphrum secundatum*.

## 4. Materials and Methods

### 4.1. Plant Material Culture

Stolons of *Stenotaphrum secundatum* were collected from field plots in the turfgrass nursery in Nanjing Botanical Garden Mem. Sun Yat-Sen, China (32.055° N, 118.834° E). The stolons with the top three nodes had these removed and cultured in water for 7 days to allow for root emergence. Then, uniform seedlings were planted in culture buckets containing 3 L of a nutrient mixture. The nutrient solutions were composed of 0.25 mM NH_4_Cl, 0.25 mM Ca(NO_3_)_2_, 0.3 mM KH_2_PO_4_, 0.35 mM K_2_SO_4_, 1 mM CaCl_2_, 1 mM MgSO_4_·7H_2_O, 20 μM EDTA-Fe, 20 μM H_3_BO_3_, 9 μM MnCl_2_·4H_2_O, 0.77 μM ZnSO_4_·7H_2_O, 0.32 μM CuSO_4_·5H_2_O, and 0.39 μM Na_2_MoO_4_·2H_2_O. The pH of the nutrient solution was 5.5. The nutrient mixture was renewed every three days. The room temperature was 28 °C, the relative humidity was 70%, the photosynthetic photon flux density was 500 μmol·m^−2^·s^−1^, and the photoperiod was 12 h/12 h (day/night). The plant material was sampled after one month of culture under these conditions.

### 4.2. Sequencing and Identification of Shaker K^+^ Channel Genes in Stenotaphrum secundatum

The sequences of the Shaker K^+^ channel genes in *Arabidopsis thaliana*, *Oryza sativa*, and *Setaria italica* were obtained from the NCBI database (https://www.ncbi.nlm.nih.gov/, accessed on 27 August 2024) (Supplementary Table S1). Since no genome database of *Stenotaphrum secundatum* was published, the transcriptome data of *Stenotaphrum secundatum* were first generated by us. The tissue used for transcriptional sequencing was mature leaves of *Stenotaphrum secundatum*, and the transcriptome data were uploaded to NCBI (PRJNA1132964, sequenced by Nuohe company, Nanjing, China). The culture conditions for the transcription sequencing of *Stenotaphrum secundatum* are described in Section 4.1.

Using the BLASTN function of TBtools software, with *Setaria italica* as a model plant, the Shaker K^+^ channel gene in the transcriptome database of *Stenotaphrum secundatum* was identified and named according to the channel type with the highest similarity [52].

### 4.3. Protein Analysis, Structure Prediction, and Three-Dimensional Modelling

The physical and chemical properties of the protein were analyzed using the online tool ProtParam (https://web.expasy.org/protparam/, accessed on 27 August 2024).

The secondary structure and visualization of the protein were performed using SOPMA (https://npsa.lyon.inserm.fr/cgi-bin/npsa_automat.pl?page=/NPSA/npsa_sopma.html, accessed on 27 August 2024) [53].

Three-dimensional protein modelling was performed using SWISS-MODEL (https://swissmodel.expasy.org/, accessed on 27 August 2024) [54].

### 4.4. Motif and Protein Domain Analysis

The MEME suite wrapper module of TBtools software was used for the motif analysis of related proteins. The number of motifs was set to 10, and the other parameters remained the default. The Pfam database was called in NCBI for the protein domain analysis. The visualization of the motif and protein domain was performed using TBtools [52,55].

### 4.5. Evolutionary Tree Construction

MEGA11 software was used to construct a phylogenetic tree of related proteins using the neighbour joining (NJ) method, with a total of 1000 bootstrap repeats. Visualization was performed in ITOL (https://itol.embl.de/, accessed on 27 August 2024) [56].

### 4.6. Construction of the SsKAT1.1-PYES2 Yeast Expression Vector and Yeast Transformation

The mature leaves of *Stenotaphrum secundatum* cultivated in Section 2.1 were cut and ground into powder. RNA was extracted using an RNA extraction kit (#RC201, vazyme, Nanjing, China) and reverse transcribed into cDNA using a reverse transcription kit (#R211, vazyme, Nanjing, China). *SsKAT1.1*-F (actataggaatattaagcttatgttcacctgcagcatata) and *SsKAT1.1*-R (tgatggatactgcagaattctactgaagaaggagaggtg) were used as primers, and cDNA was used as a template. The gene was subsequently amplified via PCR. The PCR procedure was as follows: pre-denaturation at 95 °C for 5 min, denaturation at 95 °C for 15 s, annealing at 65 °C for 15 s, extension at 72 °C for 2 min, denaturation for 30 cycles, and extension at 72 °C for 5 min. The *SsKAT1.1* gene was cloned and inserted into the pYES2 vector, and the sequence was verified by sequencing (General Biology company, Chuzhou, China).

Yeast transformation was performed in accordance with the instructions of the yeast transcription kit (#sk2400, Coolaber, Beijing, China). The yeast transformants screened in SD-URA media were verified by sequencing (General Biology, Chuzhou, China).

### 4.7. qRT-PCR

The cultivation conditions for plant materials are described as in Section 4.1. RNA was extracted from mature leaves and root tissues using an RNA extraction kit (#RC201, vazyme, Nanjing, China). The reverse transcription of RNA into cDNA was performed using a kit (#R323, vazyme, Nanjing, China). Real-time quantitative PCR (qRT-PCR) was performed using a kit as follows (#Q711, vazyme, Nanjing, China). The procedure of the qRT-PCR reaction included pre-denaturation at 95 °C for 3 min, followed by 40 cycles of 95 °C for 10 s and 60 °C for 30 s. The equipment type is BIO-RAD CFX-Opus 96. The one-way analysis of variance (ANOVA) function in SPSS 26.0 software was used for the significance analysis (*p* < 0.05). Prism9.5 software was used for graph drawing. The primers used are listed in Supplementary Table S2.

### 4.8. Subcellular Location Analysis in Tobacco

*SsKAT1.1* was constructed into a P1305 vector containing GFP and was expressed in tobacco for subcellular location experiments using an agrobacterium-mediated transformation method. The plasma membrane maker OsMCA1-RFP was simultaneously expressed in tobacco as an indicator of the position of the plasma membrane [57]. The subcellular localization of *SsKAT1.1* was observed by a laser confocal imaging analysis program (ZEISS LSM900, www.zeiss.com) [58,59].

### 4.9. Yeast Assays

The potassium ion absorption-deficient yeast R5421 was purchased from Coolaber (Beijing, China). The sequenced transformants were cultured in SD-URA liquid media supplemented with 50 mM KCl at 30 °C for 2 days. When the OD_600_ reached 0.6–0.8, the cells were collected, and the OD_600_ was adjusted to 1.0 with sterile water. The suspension was subsequently diluted by factors of 10, 100, and 1000. Five microlitres of the above suspension was spotted on AP-URA solid media supplemented with different concentrations of KCl (10, 2, 0.4, and 0.04 mM). The resulting yeast was cultured at 30 °C, and photos were taken 2 days after spotting [15].

The salt-sensitive yeast G19 was purchased from Baosai (Beijing, China). The sequenced transformants were cultured in SG-URA liquid media at 30 °C for 2 days. When the OD600 reached 0.6–0.8, the cells were collected, and the OD_600_ was adjusted to 1.0 with sterile water. The OD_600_ of the suspension was subsequently diluted to 0.1, 0.01, and 0.001 with sterile water. Five microlitres of the above suspension was spotted on AP-URA solid media supplemented with different concentrations of NaCl (0, 300, and 400 mM) and a fixed concentration of KCl (4 mM). The resulting yeast was cultured at 30 °C, and photos were taken 2 days after spotting [34].

The INVSC1 yeast used for gene cold resistance function identification was purchased from Coolaber (Beijing, China). Cold stress has been sub-divided into two types. Chilling stress is characterized by 0–15 °C, whereas temperatures below 0 °C cause freezing stress [60]; the following two types of experiments were conducted to determine the role of *SsKAT1.1* in cold resistance.

The freezing tolerance experiment: The transformants with the correct sequence were cultured in SD-URA liquid media at 30 °C for 2 days. When the OD_600_ reached 0.6–0.8, the cells were collected, and the OD_600_ was adjusted to 1.0 with SG-URA. The suspension was diluted to 0.1, 0.01, 0.001, and 0.0001 with sterile water after a pretreatment at −20 °C for 24 h. Five microlitres of the above suspension was spotted on SD-URA solid media. The resulting yeast was cultured at 30 °C, and photos were taken 2 days after spotting. The numbers of survival yeast clones at a 10,000 dilution were counted [31,61,62].

The chilling tolerance experiment: The transformants with the correct sequence were cultured in SD-URA liquid media at 30 °C for 2 days. When the OD_600_ reached 0.6–0.8, the cells were collected, and the OD_600_ was adjusted to 1.0 with sterile water. The suspensions were then diluted to 0.1, 0.01, and 0.001. Five microlitres of the above suspensions was spotted on SG-URA solid media. The resulting yeast was cultured at 30 °C and 15 °C, and photos were taken 3 days after spotting [63]. Fifty microliters (50 µL) of yeast suspension with an OD600 of 1 was diluted in 5 mL of an SG-URA liquid medium and shaken at 200 rpm at 10 °C for 6 days. The OD600 was measured every 24 h [64]. The T-test function in SPSS 26.0 software was used for the significance analysis (*p* < 0.05). The graph was generated using Prism 9.5 software.

The pH of all the yeast media was 5.8. The composition of the above media is listed in Supplementary Tables S3–S5.

## 5. Conclusions

A total of eight members of the Shaker K^+^ channel family were identified in the transcriptome of *Stenotaphrum secundatum*, the members of which are more closely related to the Shaker K^+^ channel of *Setaria italica*. *Stenotaphrum secundatum* has more *KAT1* corresponding members than other species. Yeast assays showed that *SsKAT1.1* has a potassium absorption function and has potential for cold and salt resistance in cells. The *SsKAT1.1* identified in this study provides a candidate gene resource for cultivating cold stress-resistant and multi-resistant *Stenotaphrum secundatum*.

## Figures and Tables

**Table 1:** Basic information of 9 Shaker K+ channel members of *Stenotaphrum secundatum*.

Gene Name	CDS Length	Number of Encoded Amino Acids
SsKAT1.1	2196	731
SsKAT1.2	2196	731
SsKAT2.1	1761	586
SsKAT2.2	1689	562
SsAKT1.1	2151	716
SsAKT1.2	1302	433
SsAKT2.1	2184	727
SsAKT2.2	2178	725
SsKOR1	2559	852

**Table 2:** Physicochemical properties of eight Shaker K^+^ channel member proteins in *Stenotaphrum secundatum*.

Proteins	pI	Molecular Weight (KD)	Instability Coefficient	GRAVY
SsKAT1.1	6.61	84.18	38.63	−0.223
SsKAT1.2	6.93	84.20	39.09	−0.256
SsKAT2.1	7.25	66.35	39.12	−0.247
SsKAT2.2	8.06	63.98	39.61	−0.287
SsAKT1.1	6.51	80.69	36.62	−0.231
SsAKT2.1	6.57	81.12	37.5	−0.09
SsAKT2.2	6.67	80.97	38.01	−0.09
SsKOR1	6.00	96.56	44.25	−0.123

**Table 3:** Proportion of secondary structure in 8 Shaker K^+^ channel members of *Stenotaphrum secundatum*.

Proteins	Alpha Helix (%)	Beta Turn (%)	Random Coil (%)	Extended Strand (%)
SsKAT1.1	48.43	3.15	36.11	12.31
SsKAT1.2	48.02	3.15	36.53	12.31
SsKAT2.1	49.49	2.39	35.49	12.63
SsKAT2.2	50	3.38	33.27	13.35
SsAKT1.1	47.49	7.82	37.43	7.26
SsAKT2.1	55.57	6.05	27.79	10.59
SsAKT2.2	52.83	5.66	29.52	12
SsKOR1	53.52	4.81	31.57	10.09

## Data Availability

The authors confirm that the data supporting the findings of this study are available within the article.

## Supplementary Materials

The following supporting information can be downloaded at: https://www.mdpi.com/article/10.3390/ijms25179480/s1.

## Abbreviations

**Note:** UDP-glucuronosyltransferases (*UGTs*), 9-cis epoxy carotenoid dioxygenase (*NCED*), raffinose synthetase (*RAF*), C-repeat binding factor (*CBF*), dehydration-responsive element binding protein (*DREB*)
