# Supplementary material for: Identification of Shaker Potassium Channel Family Members and Functional Characterization of SsKAT1.1 in Stenotaphrum secundatum Suggest That SsKAT1.1 Contributes to Cold Resistance
Source: Int J Mol Sci. 2024 Aug 31;25(17):9480. doi: 10.3390/ijms25179480 (PMC11394884; doi:10.3390/ijms25179480)
Supplement: Supplementary file 1 [file ijms-25-09480-s001.zip › ijms-3147234-supplementary.pdf]

Identification of Shaker Potassium Channel Family Members and Functional Characterization of *SsKAT1.1* in *Stenotaphrum secundatum* Suggest That *SsKAT1.1* Contributes to Cold Resistance

Dong-Li Hao [1,†], Jia Qu [2,†],Zhi-Yong Wang [2], Dao-Jin Sun [1], Sheng-Nan Yang [2], Jian-Xiu Liu [1], Jun-Qin Zong [1,*] and Hai-Long Lu [1,*]

Supplementary Table S1. Gene names and ID numbers of Shaker K+ channels.

**Arabidopsis thaliana**		**Oryza sativa**		**Setaria italica**		**Zea mays**	
gene	ID	gene	ID	gene	ID	gene	ID
AtKAT1	AT5G46240	OsKAT1	LOC4328864	SiKAT1	LOC101784878	ZmKAT1	LOC103626996
AtKAT2	AT4G18290	OsKAT2	LOC4325560	SiKAT2	LOC101769500		
AtKAT3	AT4G32650	OsKAT3	LOC4325143	SiKAT3	LOC101758676		
AtKT1	AT2G26650	OsKAT4	LOC107276707	SiKAT4	LOC101765194		
AtKT2/3	AT4G22200	OsKAT6	LOC107276718	SiKAT6	LOC101752735		
AtKT5	AT4G32500	OsAKT1	LOC4326245	SiAKT1	LOC101757319		
AtSPIK	AT2G25600	OsAKT2	LOC4338867	SiAKT2	LOC101772930		
AtGORK	AT5G37500	OsAKT3	LOC9271501	SiAKT3	LOC101770233		
AtSKOR	AT3G02850	OsKOR1	LOC4340653	SiKOR1	LOC101767716		
		OsKOR2	LOC4335955	SiKOR2	LOC101765339		

Supplementary Table S2. Primers used for qRT-PCR.

Name	Primer sequence (5′-3′)
ACTIN-F	CAAGAACTACGACCCGCAAA
ACTIN-R	AGCCTCGGAAGCTAAGAAAG
SsKAT1.1-F	TCTCGAGAAGCGTGGTTGTT
SsKAT1.1-R	CCCACCTGTTGCCCTGTAAT
SsKAT1.2-F	AATGGAAGTGCAGGCTGAGT
SsKAT1.2-R	AGCACCAACTTCCCCAAACA
SsKAT2.1-F	TGCTTGCATTCTCGGTGGAT
SsKAT2.1-R	TGAGCAGTTTCGTGGGACAA

SsKAT2.2-F	TTGGTGAAATCGGGGCTCTC
SsKAT2.2-R	ATTTCCCCGTGAGCTCAGTG
SsAKT1.1-F	CCGAACGTGGACCATGAAGA
SsAKT1.1-R	CCTGACGAGGGAGTTGTGAC
SsAKT2.1-F	ATCGGCAACATGACCAACCT
SsAKT2.1-R	CTCTCCGCCCTGAACTTGAG
SsAKT2.2-F	GGCCTCGGGTGAGCATATAC
SsAKT2.2-R	TGTCCCGGATCACGTCAATG
SsKOR1-F	GGTGAAGACGGCCAAGAAGA
SsKOR1-R	GGTGAAGGACTGCTTGTCGA

Supplementary Table S3. Composition of SD-URA.

Reagent	Contains per 100 ml
glucose	2 g
DO Supplement-Ura (#PM2270, coolaber, Beijing, China)	0.07 g
YNB (without AA, # PM2070, coolaber, Beijing, China,)	0.67 g
Agarose (Liquid medium is not added)	2 g
KCl	The amount of addition depends on the requirements in the text

NOTE：AA：Amino Acid

Supplementary Table S4. Composition of SG-URA.

Reagent	Contains per 100 ml
galactose	2 g
DO Supplement-Ura (#PM2270, coolaber, Beijing, China)	0.07 g
YNB (without AA, #PM2070, coolaber, Beijing, China)	0.67 g
Agarose (Liquid medium is not added)	2 g
KCl	The amount of addition depends on the

	requirements in the text

Supplementary Table S5. Composition of AP-URA.

Reagent	Contains per 100 ml
galactose	2 g
DO Supplement-Ura(#PM2270,coolaber,China ,beijin)	0.07 g
5×AP liquid Medium (#PM4060L,coolaber,China,beijin)	20 ml
Agarose(Liquid medium is not added)	2 g
KCl	The amount of addition depends on the requirements in the text
NaCl	The amount of addition depends on the requirements in the text
